# LC-MS Supported Studies on the *in Vitro* Metabolism of both Enantiomers of Flubatine and the *in Vivo* Metabolism of (+)-[^18^F]Flubatine—A Positron Emission Tomography Radioligand for Imaging α4β2 Nicotinic Acetylcholine Receptors

**DOI:** 10.3390/molecules21091200

**Published:** 2016-09-08

**Authors:** Friedrich-Alexander Ludwig, René Smits, Steffen Fischer, Cornelius K. Donat, Alexander Hoepping, Peter Brust, Jörg Steinbach

**Affiliations:** 1 Helmholtz-Zentrum Dresden-Rossendorf, Research Site Leipzig, Institute of Radiopharmaceutical Cancer Research, Permoserstraße 15, Leipzig 04318, Germany; f.ludwig@hzdr.de (F.-A.L.); s.fischer@hzdr.de (S.F.); j.steinbach@hzdr.de (J.S.); 2 ABX Advanced Biochemical Compounds GmbH, Heinrich-Gläser-Straße 10-14, Radeberg 01454, Germany; smits@abx.de (R.S.); hoepping@abx.de (A.H.); 3 Division of Brain Sciences, Imperial College London, Du Cane Road, London W12 0NN, UK; c.donat@imperial.ac.uk

**Keywords:** nicotinic acetylcholine receptors (nAChRs), epibatidine, flubatine, NCFHEB, positron emission tomography (PET), radiometabolites, liquid chromatography-mass spectrometry (LC-MS), liver microsomes

## Abstract

Both enantiomers of [^18^F]flubatine are promising radioligands for neuroimaging of α4β2 nicotinic acetylcholine receptors (nAChRs) by positron emission tomography (PET). To support clinical studies in patients with early Alzheimer’s disease, a detailed examination of the metabolism *in vitro* and *in vivo* has been performed. (+)- and (−)-flubatine, respectively, were incubated with liver microsomes from mouse and human in the presence of NADPH (β-nicotinamide adenine dinucleotide 2′-phosphate reduced tetrasodium salt). Phase I *in vitro* metabolites were detected and their structures elucidated by LC-MS/MS (liquid chromatography-tandem mass spectrometry). Selected metabolite candidates were synthesized and investigated for structural confirmation. Besides a high level of *in vitro* stability, the microsomal incubations revealed some species differences as well as enantiomer discrimination with regard to the formation of monohydroxylated products, which was identified as the main metabolic pathway in this assay. Furthermore, after injection of 250 MBq (+)-[^18^F]flubatine (specific activity > 350 GBq/μmol) into mouse, samples were prepared from brain, liver, plasma, and urine after 30 min and investigated by radio-HPLC (high performance liquid chromatography with radioactivity detection). For structure elucidation of the radiometabolites of (+)-[^18^F]flubatine formed *in vivo*, identical chromatographic conditions were applied to LC-MS/MS and radio-HPLC to compare samples obtained *in vitro* and *in vivo*. By this correlation approach, we assigned three of four main *in vivo* radiometabolites to products that are exclusively *C*- or *N*-hydroxylated at the azabicyclic ring system of the parent molecule.

## 1. Introduction

Positron emission tomography (PET) is a non-invasive imaging technique that enables a quantitative space- and time-dissolved assessment of biochemical, physiological and pathophysiological processes in human [1]. For the imaging of enzyme or receptor systems, selectively binding radiotracers, carrying a short-lived positron-emitting nuclide, e.g., ^11^C (t_1/2_ = 20.4 min) or ^18^F (t_1/2_ = 109.8 min), are required [2].

**Figure 1:**
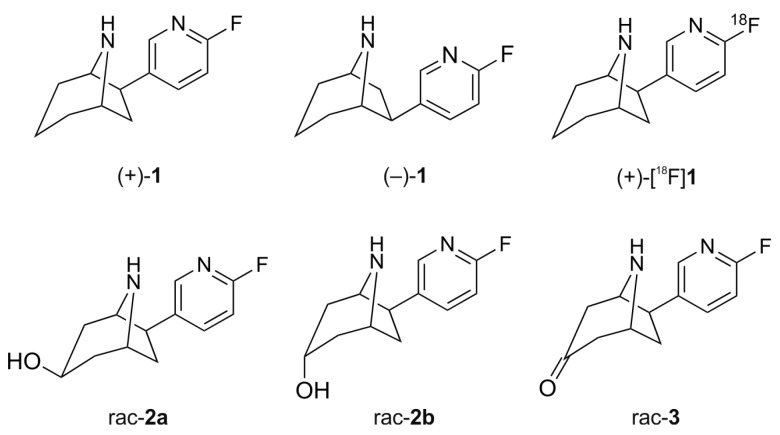
Chemical structures of (+)-flubatine ((+)-**1**), (−)-flubatine ((−)-**1**), (+)-[^18^F]flubatine ((+)-[^18^F]**1**) and reference compounds (±)-*exo*-3-hydroxy-flubatine (*rac*-**2a**), (±)-*endo*-3-hydroxy-flubatine (*rac*-**2b**), (±)-flubatine-3-one (*rac*-**3**).

The PET radioligands (+)-[^18^F]flubatine ((+)-[^18^F]**1**, Figure 1) and (−)-[^18^F]flubatine have been derived from (−)-epibatidine ((1*S*,2*R*,4*R*)-*exo*-2-(6-chloro-3-pyridyl)-7-azabicyclo[2.2.1]heptane), which has been isolated from the skin of the Ecuadorian poison dart frog *Epipedobates anthonyi* [3]. Structural modifications of this alkaloid led to derivatives with lower toxicity [4,5,6], and in particular to the two norchloro-fluoro-homoepibatidine enantiomers, namely (+)- and (−)-flubatine. For both, a NOEL (No Observed Effect Level) of 1.55 μg/kg and 6.2 μg/kg, respectively, was calculated for human application [7]. Moreover, due to their high affinity and specificity towards α4β2 nAChRs [8], which are involved in the pathogenesis of Alzheimer’s Disease and other neurological disorders [9,10], both [^18^F]flubatine enantiomers have been developed for imaging of these receptors with PET [7,11,12,13,14]. (−)-[^18^F]Flubatine has already been evaluated in rhesus monkeys [15,16,17] and in human [18,19,20,21], and very recently, also (+)-[^18^F]flubatine has been investigated in a first clinical study [22].

To support clinical studies, determination of the fraction of unmetabolized radioligand in plasma related to formed radioactive metabolites is needed to deliver an arterial input function for quantitative PET measurement [23]. Due to very low concentrations within a picomolar to nanomolar range, only radiodetection, e.g., by radioactivity flow detectors coupled to HPLC (high performance liquid chromatography), can be used to detect molecular moieties bearing the positron-emitting nuclide. PET measurements cannot distinguish signals which result from different chemical species. The occurrence, the binding properties and the distribution of metabolites of the radioligand (radiometabolites) might have tremendous impact on quality and reliability of PET images. To identify potential radiometabolites, non-labeled references of the radioligands are examined in animal models as well as *in vitro*. Subsequent investigations by LC-MS (liquid chromatography-mass spectrometry) enable the structural elucidation of formed metabolites [24,25,26,27,28].

The design of these studies on both flubatine enantiomers was based on reported data for epibatidine and the identification of its metabolites by mass spectrometry [29,30,31]. Those *in vitro* studies on liver microsomes revealed significant species differences. Furthermore, routes of metabolism were identified for both epibatidine enantiomers, namely hydroxylation of the azabicyclic ring and formation of *N*-oxides.

Initially, we examined (+)- and (−)-flubatine concerning the oxidative metabolism by cytochrome P450 enzymes using mouse (MLM) and human (HLM) liver microsomes. Besides structural elucidation of *in vitro* metabolites by LC-MS/MS (liquid chromatography-tandem mass spectrometry), we synthesized selected reference compounds (Figure 1) to enable an unequivocal assignment. Although radio-HPLC (high performance liquid chromatography with radioactivity detection) cannot deduce structural information on radiometabolites as LC-MS/MS techniques, radiochromatograms provide valuable information as they represent the complete pattern of formed radiometabolites as well as their relative amounts. Therefore, the fraction of unchanged radioligand and of any radiometabolite can be determined easily with satisfactory correctness. Using this advantage, we investigated radiolabeled (+)-[^18^F]flubatine in mice to detect radiometabolites *in vivo*. Finally, we identified structures of several radiometabolites by correlation with results obtained *in vitro*.

## 2. Results and Discussion

### 2.1. Synthesis of Reference Compounds

**Figure 2:**
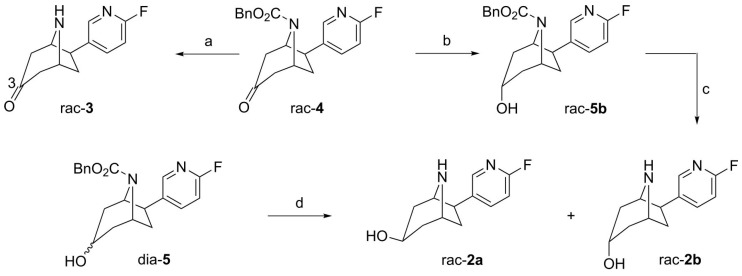
Synthesis of reference compounds. *Conditions*: (**a**) Pd/C (10%), cyclohexene, EtOH, reflux, 16 h, 59%; (**b**) 1. l-Selectride, THF, −78 °C, 2 h; 2. NaOH, H_2_O_2_, H_2_O, THF, room temperature (rt), 1 h, 97%; (**c**) Pd/C, cyclohexene, EtOH, reflux, 17 h, 28%; and (**d**) HBr (33% in AcOH), Et_2_O, 0 °C, 5 h, 23% *rac*-**2a**, 34% *rac*-**2b**.

To support structural elucidation of metabolites formed *in vitro*, the 3-hydroxy and 3-keto reference compounds (*rac*-**2a**, *rac*-**2b** and *rac*-**3**) were synthesized starting from *rac*-**4** and *dia*-**5** (Figure 2) [7]. Removal of the carboxybenzyl (Cbz) protecting group of *rac*-**4** under transfer hydrogenation conditions gave *rac*-**3**. For the synthesis of the C3 *endo* alcohol *rac*-**2b** l-Selectride (lithium tri-*sec*-butylborohydride) was used as reducing agent. The stereospecific reduction of 8-aza-bicyclo[3.2.1]octan-3-ones with l-Selectride is known from the literature [32]. After reduction, *rac*-**5b** was deprotected to afford the diastereomerically pure C3 *endo* alcohol *rac*-**2b**. Cbz deprotection of *dia*-**5**, obtained by unspecific ketone reduction with sodium borohydride (1.3:1 diastereomeric mixture), gave the epimeric alcohols *rac*-**2a** and *rac*-**2b**. The desired C3 *exo* alcohol *rac*-**2a** could be isolated by column chromatography.

### 2.2. Investigation of Microsomal Incubations by LC-MS

In order to identify metabolites and to compare conversions corresponding to phase I metabolism, both, (+)- and (−)-**1**, were incubated with liver microsomes from mouse and human (MLM and HLM, respectively), in the presence of NADPH (β-nicotinamide adenine dinucleotide 2′-phosphate reduced tetrasodium salt), at 37 °C for 90 min.

Samples were examined regarding multiple reaction monitoring (MRM) transitions, deduced from the enhanced product ion (EPI) spectrum of *rac*-**1**. To complete the selection, the metabolite identification software LightSight (Verson 2.3.0.152038, AB SCIEX, Darmstadt, Germany) was used. No substitution or elimination of fluorine could be detected. To identify potential oxidation products, changes of *m*/*z* +14, +16, +28, +30, +32, and +48 with respect to the parent molecule, were studied measuring appropriate MRM transitions. Starting from the intense MRM transition of 207.1/110.0 found for *rac*-**1**, oxidation products showing an analogous MRM transition of 223.1/110.0 have been detected. This corresponds to hydroxylation of flubatine, which was found to be the major biotransformation reaction under the conditions described herein, whilst other oxidative processes appeared to be negligible.

**Figure 3:**
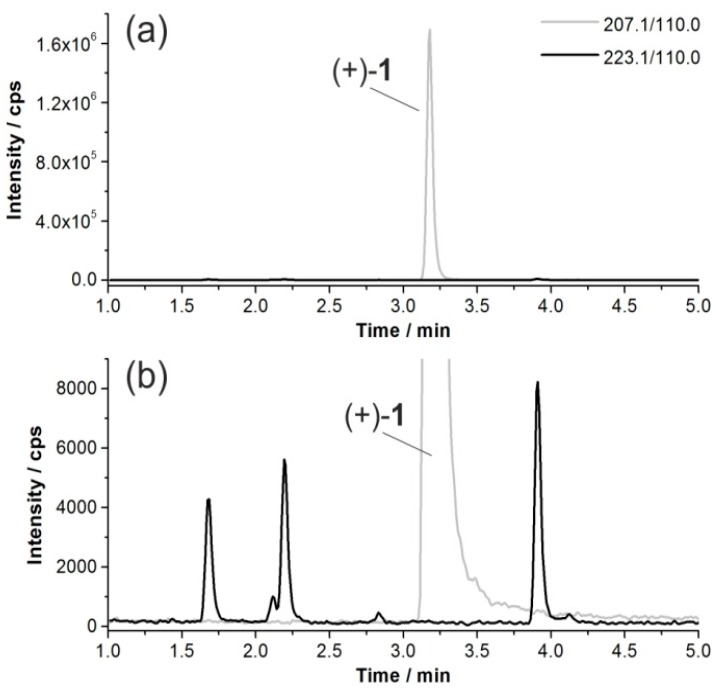
(**a**) Multiple reaction monitoring (MRM) chromatograms of an acetonitrile extract after incubation of (+)-**1** with mouse liver microsomes (MLM) (37 °C, 90 min), where ion traces represent unconverted (+)-**1** (*m*/*z* 207.1 [M_parent_ + H]^+^, *t*_R_ = 3.2 min) and hydroxylated products (*m*/*z* 223.1 [M_parent_ + O + H]^+^) according to the MRM transitions indicated; and (**b**) enlarged detail of (**a**).

Since the fragment ion of *m*/*z* 110.0 can be attributed to an unaffected 2-fluoro-pyridyl moiety, only hydroxylations at the azabicyclic ring system had to be considered. However, our investigations were challenged by the low extent of metabolization. Figure 3 shows MRM chromatograms of a sample obtained after incubation of (+)-**1** with MLM.

### 2.3. In Vitro Formation of Monohydroxylated Metabolites of *(+)-**1*** and *(−)-**1***

**Figure 4:**
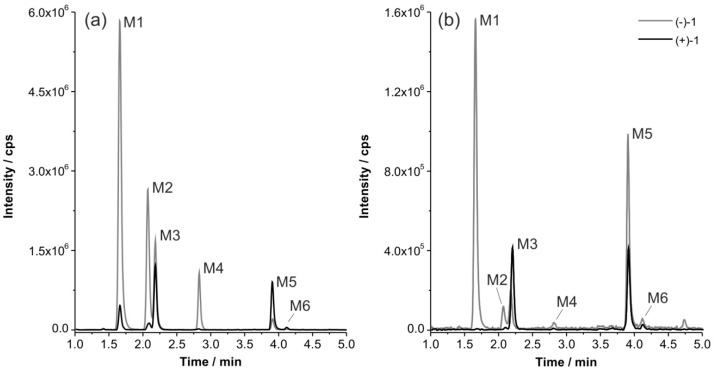
Q3 multiple ion (MI) chromatograms for *m*/*z* 223.1 representing monohydroxylated products of the parent flubatine enantiomers after incubation with: (**a**) mouse liver microsomes (MLM); and (**b**) human liver microsomes (HLM) (37 °C, 90 min each).

In order to compare their tendency for oxidation by MLM and HLM, we examined the conversion of (+)- and (−)-**1** using a Q3 multiple ion (MI) scan mode, monitoring ions with a *m*/*z* of 223.1, which corresponds to [M_parent_ + O + H]^+^ (Figure 4). In general, a high level of stability was preserved for all incubations. Both flubatine enantiomers were metabolized with higher extent during incubation with MLM than with HLM, whilst (+)-**1** was even more stable than (−)-**1**. A series of metabolites M1 to M6 was detected, eluting at 1.7 min, 2.1 min, 2.2 min, 2.8 min, 3.9 min, and 4.1 min, respectively.

Metabolites from incubations of (+)- and (−)-**1** with identical retention times were considered as enantiomeric metabolites due to achiral HPLC conditions. This was supported by EPI spectra that showed similar fragmentation patterns in terms of detected fragment ions and intensities. For discussion, enantiomeric metabolites are referred to as, e.g., M1 instead of (+)-M1 or (−)-M1.

With both mouse and human microsomes (MLM and HLM), M1 was formed as major metabolite of (−)-**1**. By contrast, for (+)-**1**, M1 was detected only after incubation with MLM but not formed in presence of HLM. M4 was detected with considerable intensity only after incubation of (−)-**1** with MLM. M3 and M5 were mainly formed when (+)-**1** was incubated with HLM or MLM.

### 2.4. Structure Elucidation of Monohydroxylated in Vitro Metabolites by MS/MS

**Figure 5:**
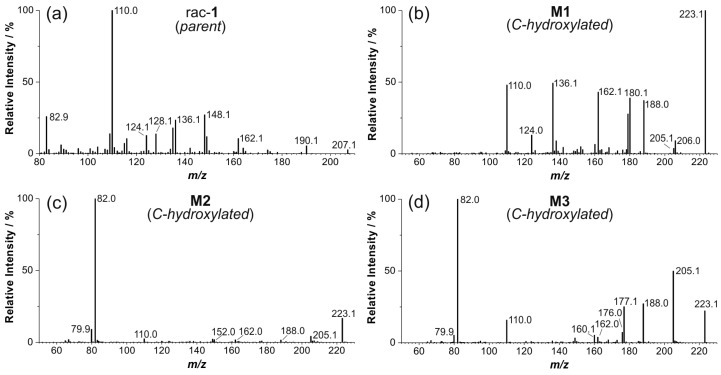

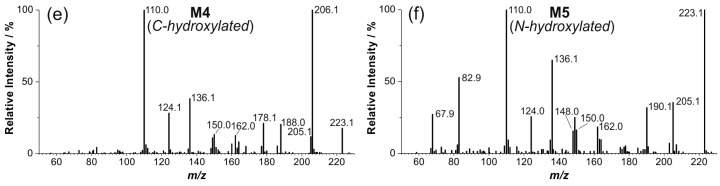
Enhanced product ion (EPI) spectra of: (**a**) *rac*-**1** (product of *m*/*z* 207.1, CE 70, CES 25); and (**b**–**f**) metabolites M1 to M5 (products of *m*/*z* 223.1, CE 33, CES 0).

**Figure 6:**
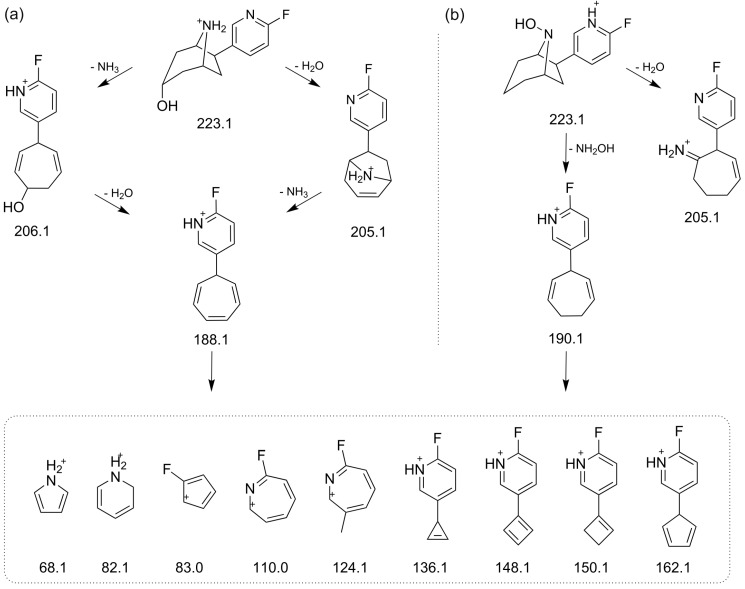
Proposed basic fragmentation pathways and structures for observed major fragment ions of metabolites detected during enhanced product ion (EPI) scans: (**a**) for M0 to M4 (illustrated for M1); and (**b**) for M5 (occurrence and intensity of fragments vary depending on the respective metabolite, and regiochemical, stereochemical and mesomeric issues have been neglected).

The EPI spectrum of *rac***-1** shows a neutral loss of *m*/*z* 17, due to elimination of ammonia, whereas the intense signal at *m*/*z* 110.0 represents a 2-fluoro-azatropylium ion originating from the 2-fluoro-pyridyl moiety (Figure 5). Since this signal was found for M1 to M6, evidence was given that hydroxylations have taken place at the azabicylic ring system only, but not at the pyridyl site. For M1 to M4, eliminations of ammonia (*m*/*z* 17) and water (*m*/*z* 18) led to signals at *m*/*z* 206.1, 205.1, and 188.1. While elimination of ammonia resulted from an unsubstituted NH-group, elimination of water further confirmed a *C*-hydroxylation at the bicyclic part of M1 to M4, respectively. During fragmentation, both elimination processes result in protonated cycloheptatrien structures (*m*/*z* 188.1) (Figure 5b–e and Figure 6a). By contrast, M5 lacks the aforementioned product ion and the signal at *m*/*z* 190.1 points to an elimination of hydroxyl amine (*m*/*z* 33) due to a hydroxylation at the bridgehead nitrogen atom (Figure 5f and Figure 6b). For M6 no EPI spectrum with sufficient quality could be recorded, due to its low concentration. However, significant fragments, e.g., *m*/*z* 110.0 and 190.1, were detected for M6 and for the *N*-hydroxylated metabolite M5.

### 2.5. Structural Identification of Monohydroxylated in Vitro Metabolites by Aid of Reference Compounds

**Figure 7:**
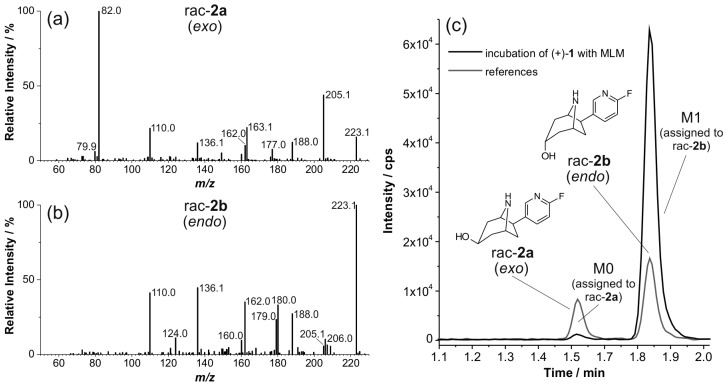
Enhanced product ion (EPI) spectra of: (**a**) *rac*-**2a**; and (**b**) *rac*-**2b** (products of *m*/*z* 223.1, CE 33, CES 0). (**c**) Details of multi reaction monitoring (MRM) chromatograms (223.1/110.0) of an extract resulting from incubation of (+)-**1** with mouse liver microsomes (MLM) (37 °C, 90 min) and a mixture of the references *rac*-**2a** and *rac*-**2b**.

**Figure 8:**
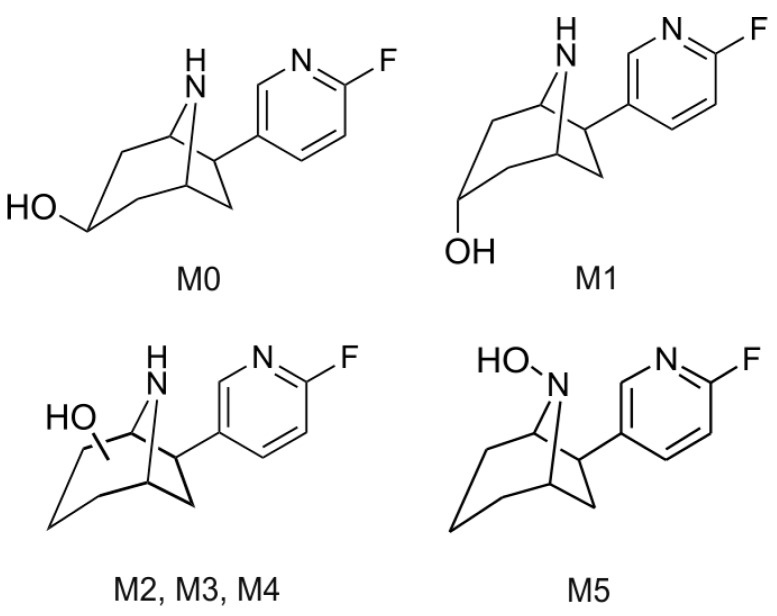
Identified monohydroxylated metabolites of (+)-**1** after incubation with liver microsomes.

To elucidate the chemical structures of the *C*-hydroxylated metabolites M1 to M4, comparisons were made with reference compounds synthesized as described above. We assumed that the C3 *exo* and *endo* alcohols *rac*-**2a** and *rac*-**2b** (Figure 1) could be relevant candidates. Based on the identical LC retention time and matching EPI spectra (Figure 7b,c), the metabolite M1 was assigned to the *endo* epimer *rac*-**2b**. Further analysis of extracts from microsomal incubations of (+)-**1** with MLM revealed another metabolite (M0). Due to low concentrations of M0, no EPI spectrum of sufficient quality could be recorded. However, the pattern of significant fragment ions and the LC retention time match with that of reference *rac*-**2a**, thus enabling an assignment of M0 (Figure 6a and Figure 7a,c). Figure 8 summarizes the monohydroxylated metabolites of (+)-**1** that were identified.

The formation of monohydroxylated products *in vitro* has also been previously reported for the epibatidine enantiomers [29]. As described above, only the azabicyclic ring of the flubatine enantiomers (+)-**1** and (−)-**1** was affected as it has been reported for epibatidine. Up to seven metabolites were detected when (+)-**1** or (−)-**1** were incubated with MLM. For HLM, the patterns of metabolites were similar but showed some differences. For instance, incubation of (+)-**1** with HLM only resulted in two main metabolites, which appears to be in contrast to the fate of epibatidine as no metabolites could be detected after incubations with HLM as well as liver microsomes from rat [29]. However, up to six metabolites were formed by incubations with liver microsomes from rhesus monkey and dog [29]. It might be the case that the MS method used for *in vitro* studies of epibatidine was not sensitive enough to detect metabolites at very low concentrations. Further, by means of synthesized reference compounds, the structures of some of the *C*-hydroxylated flubatine metabolites could be elucidated or assigned more precisely. As deduced for epibatidine, oxidation by cytochrome P450 enzymes took place even at the nitrogen atom of the azabicyclic ring system. Since secondary amines are known to be exclusively oxidized to hydroxylamines [33], we concluded an *N*-hydroxylation of both flubatine enantiomers, even though the term “*N*-oxides” was used in the literature for corresponding products of epibatidine [29].

### 2.6. Dihydroxylated Products and Ketones Formed in Vitro

We obtained clear evidence for dihydroxylation and ketone formation *in vitro* that occurred to an even lower extent than monohydroxylation.

**Figure 9:**
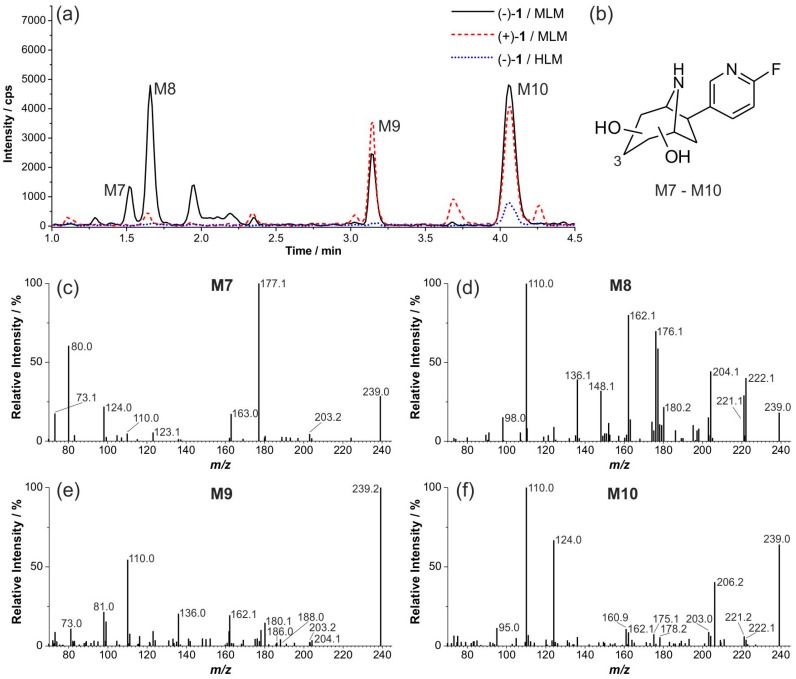
Formation of dihydroxylated metabolites: (**a**) multi reaction monitoring (MRM) chromatograms (239.1/110.0) of extracts resulting from incubations of both (+)-**1** and (−)-**1** with liver microsomes (37 °C, 90 min); (**b**) general formula of dihydroxylated metabolites of (+)-**1**; and (**c**–**f**) enhanced product ion (EPI) spectra of M7 to M10 (products of *m*/*z* 239.1, CE 33, CES 0).

Incubations of (+)-**1** and (−)-**1** with MLM and of (−)-**1** with HLM resulted in dihydroxylated products as shown by the MRM chromatograms in Figure 9a. Peaks are labeled (M7–M10) if EPI spectra of sufficient quality could be recorded (Figure 9c–f). For M7 to M10 (Figure 9b), a twofold neutral loss of 18 was found, indicating eliminations of water resulting from hydroxyl groups. For the metabolite M8, the fragment ion of *m*/*z* 222.1 that results from elimination of ammonia (*m*/*z* 17) provides evidence that no *N*-hydroxylation but a twofold *C*-hydroxylation occurred. The EPI spectrum of M9 shows signals that might represent a twofold neutral loss of water (*m*/*z* 18), followed by elimination of ammonia (*m*/*z* 17). Furthermore, the signal at *m*/*z* 188.0 probably arises from eliminations of hydroxylamine (*m*/*z* 33) and water (*m*/*z* 18) and points to a *C*/*N*-dihydroxylation. We conclude that the more hydrophobic metabolite M10 is another *C*/*N*-dihydroxylated product, since the signal at *m*/*z* 206.2 strongly indicates an elimination of hydroxylamine (*m*/*z* 33) from the parent ion.

**Figure 10:**
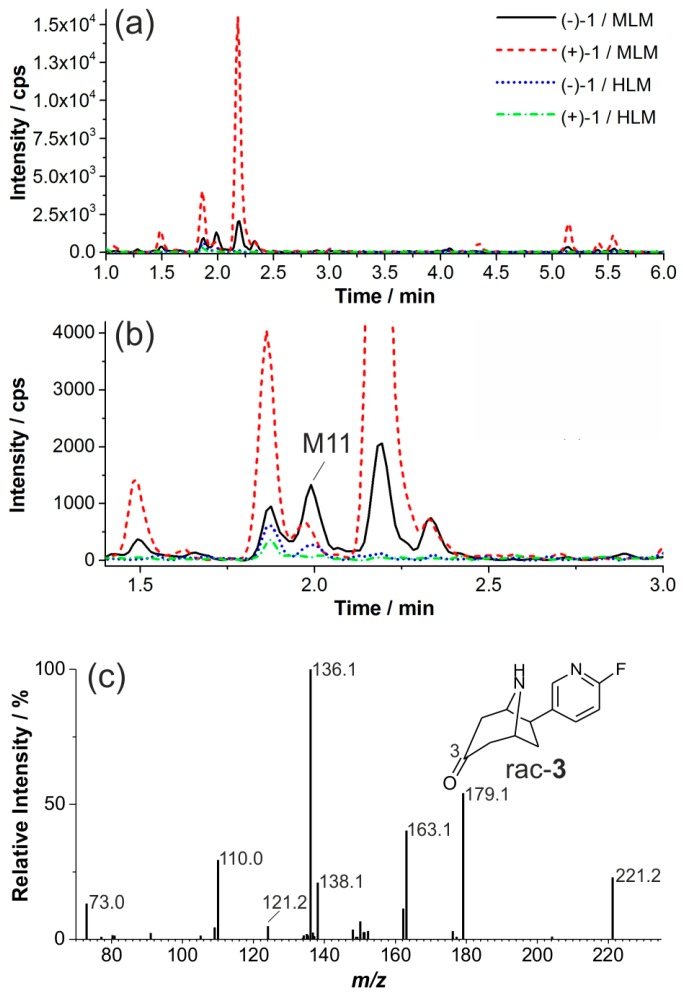
Formation of ketones: (**a**) multi reaction monitoring (MRM) chromatograms (222.1/110.0) of extracts resulting from incubations of both (+)-**1** and (−)-**1** with liver microsomes (37 °C, 90 min); (**b**) enlarged detail of (**a**); and (**c**) enhanced product ion (EPI) spectrum of *rac-***3** (product of *m*/*z* 221.1, CE 33, CES 0).

Next, metabolites with an MRM transition of 221.1/110.0 were investigated (Figure 10). Conversions of (−)-**1** and (+)-**1** by incubation with HLM were negligible, whilst some metabolites were formed by incubation with MLM (Figure 10a,b). Incubations of both 3-hydroxy epimers *rac*-**2a** and *rac*-**2b** gave the same product, eluting at 2.0 min. Retention time and EPI spectra are in accordance with data obtained from reference *rac*-**3** and metabolite M11, which thereby was identified as flubatine-3-one. For the remaining metabolite, pattern structures could not be elucidated further.

Dihydroxylation and formation of ketones from both flubatine enantiomers, (+)-**1** and (−)-**1**, only played a limited role for the metabolism *in vitro*. For both enantiomers of epibatidine, these metabolic pathways have not been found, perhaps because of the sensitivity of the mass detector used [29].

### 2.7. Investigation of the in Vivo Metabolism of *(+)-[^18^F]**1***

In order to obtain radiometabolites formed *in vivo*, (+)-[^18^F]**1** was injected into a CD-1 mouse. At 30 min p.i., plasma and urine samples, as well as liver and brain homogenates were extracted using ice-cold acetonitrile. Recoveries of 92%–95% (*N* = 2–8) were achieved. Samples were analyzed by radio-HPLC. The method (method III) applied herein is based on conditions described in literature for the separation of *in vitro* metabolites of epibatidine [29]. We used the following stationary phases: Reprosil-Pur C18-AQ, Multospher 120 RP 18 AQ-5 μ, Prodigy 5 μ C8 (250 mm × 4.0 mm or 4.6 mm, and 5 μm, respectively) as well as different solvent systems for gradient elution: ammonium acetate (20 mM, pH 7.4)/acetonitrile, ammonium formate (25 mM, pH 3)/acetonitrile and water/acetonitrile/methanol (0.2% TFA). Besides the Prodigy C8, the Multospher RP 18 AQ-column appeared most suitable when ammonium formate (pH 3) and acetonitrile were used as mobile phase.

Non-metabolized (+)-[^18^F]**1** still represented more than 93% of the radioactive substances in the extract obtained from mouse brain at 30 min p.i., which supports the suitability of this radioligand for clinical applications.

**Figure 11:**
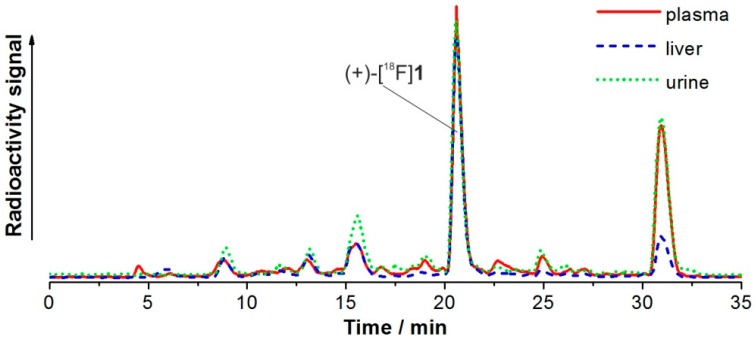
Radiochromatograms (method III) of acetonitrile extracts after injection of (+)-[^18^F]**1** in CD-1 mouse (30 min p.i.).

Two radiometabolites (<7% in total) detected in brain were also found in plasma, liver, and urine, where they represented the main fractions, respectively (*t*_R_ = 15.5 min: 7% (plasma), 12% (liver), 12% (urine); *t*_R′_ = 30.9 min: 29%, 11%, 28%). The radiometabolite patterns were similar for all extracts and the highest level of metabolites was determined in urine (Figure 11). Altogether, up to 17 radiometabolites could be detected by radio-HPLC. Most radiometabolites were more polar than the parent tracer. However, the major metabolite eluted after a longer retention time (30.9 min) than (+)-[^18^F]**1**.

### 2.8. Studies on Identification of Radiometabolites of *(+)-[^18^F]**1***

**Figure 12:**
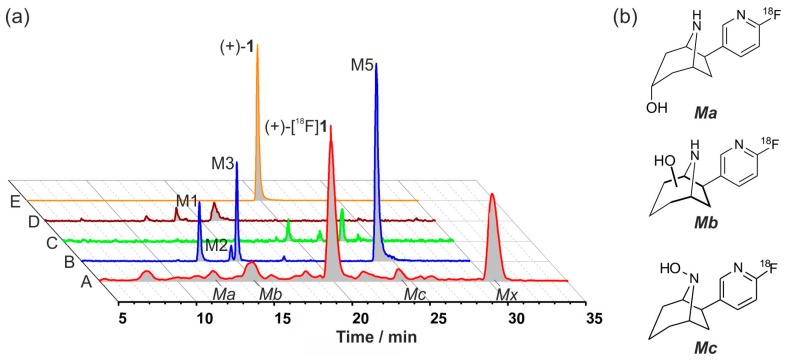
Identification of radiometabolites: (**a**) Aligned radio-HPLC and LC-MS chromatograms (method II). A, radioactivity signal: (+)-[^18^F]**1** in mouse (plasma, 30 min p.i.); and multi reaction monitoring (MRM) chromatograms (*m*/*z* parent/product ion) after incubation of (+)-**1** with mouse liver microsomes (MLM) (37 °C, 90 min); B, monohydroxylation (223.1/110.0); C, dihydroxylation (239.1/110.0); D, ketone formation (221.1/110.0); and E, (+)-**1** (207.1/110.0). Signal intensities have been adapted; (**b**) Structures of assigned radiometabolites.

LC-MS analyses served to elucidate the chemical structures of radiometabolites formed in mice. For that purpose, the extract obtained from MLM incubation of (+)-**1** was measured by LC-MS/MS under the same LC conditions (method II) as for radio-HPLC. On the LC-MS (+)-**1** eluted 0.8 min earlier than (+)-[^18^F]**1** on the radio-HPLC system, despite identical chromatographic conditions. Therefore, radiochromatograms were normalized to the retention time of (+)-**1** (*t*_R_ = 19.8 min) on the LC-MS system. Figure 12a compares the radiochromatogram obtained from mouse plasma after administration of (+)-[^18^F]**1** (radioactivity signal, A) and MRM chromatograms of monohydroxylated (B), dihydroxylated (C), ketone (D) metabolites as well as unchanged (+)-**1** (E) after its incubation with MLM. The radiometabolite *Ma* matched the identified *in vitro* metabolite M1 and was assigned as [^18^F]3-*endo*-hydroxy-flubatine. Matches were also found for *Mb* and *Mc*, which were assigned to *C*-hydroxylated flubatine (M2/M3) and *N*-hydroxylated flubatine (M5), respectively. The structures of the identified radiometabolites are shown in Figure 12b. A precise assignment of further radiometabolites was not possible. However, for some products detected by radio-HPLC dihydroxylation and ketone formation can be considered. For the major radiometabolite *Mx*, no adequate *in vitro* metabolite has been found so far. It can be assumed that this radiometabolite results from phase II metabolism of (+)-[^18^F]**1**.

## 3. Experimental Section

### 3.1. Chemicals and Reagents

Solvents for synthesis were purchased from Merck (Darmstadt, Germany) and Fisher Scientific (Schwerte, Germany). Chemicals were obtained from Merck, Fisher Scientific, Sigma-Aldrich (Steinheim, Germany), C. Roth (Karlsruhe, Germany) and Machery-Nagel (Düren, Germany). All chemical reagents were of highest commercially available quality and applied without further purification. (+)-**1**, (−)-**1**, *rac*-**4**, and *dia*-**5** were synthesized as previously reported [7]. Acetonitrile (gradient grade) was purchased from VWR International (Darmstadt, Germany). Acetonitrile and water (both for LC-MS) were purchased from Fisher Scientific. Ammonium formate (for HPLC) was purchased from Acros Organics (Geel, Belgium). Formic acid and ammonium formate (both LC-MS), testosterone, and NADPH were purchased from Sigma-Aldrich. GIBCO mouse liver microsomes (MLM, 20 mg/mL) and GIBCO human liver microsomes (HLM, pooled donors, 20 mg/mL) were purchased from Life Technologies (Darmstadt, Germany). Dulbecco’s phosphate buffered saline (PBS) (without Ca^2+^, Mg^2+^) was purchased from Biochrom (Berlin, Germany).

### 3.2. Synthesis of Reference Compounds

For reaction monitoring, thin-layer chromatography (TLC) was performed with Macherey-Nagel plastic sheets precoated with fluorescent indicator UV254 (Polygram SIL G/UV254). Visualization of the spots was effected by irradiation with an UV lamp (254 nm and 366 nm). ^1^H Nuclear magnetic resonance (NMR) spectra were obtained with a Bruker AV500 spectrometer (Bruker Corporation, Billerica, MA, USA). Chemical shifts are reported as δ values. Coupling constants are reported in Hertz. Electrospray ionization (ESI) mass spectra were obtained using a Surveyor MSQ Plus mass detector (Thermo Fisher Scientific GmbH, Dreieich, Germany).

#### 3.2.1. (±)-*exo*-6-(6-Fluoro-pyridin-3-yl)-8-aza-bicyclo[3.2.1]octan-3-one ((±)-flubatine-3-one, *rac*-**3**)

*rac*-**4** (300 mg, 0.85 mmol) was stirred in cyclohexene (4.1 mL) and ethanol (8.5 mL) until complete dissolution. The solution was placed under argon and 10% palladium on activated carbon (Pd/C) (178.5 mg, 0.17 mmol, 0.2 eq.) was added carefully. The reaction mixture was heated to reflux for 16 h. After cooling to room temperature (rt), the reaction mixture was filtered over celite. The filtration residue was washed with methanol and the solvent was removed *in vacuo*. The crude product was purified by column chromatography (methanol:ethyl acetate = 1:10) to afford *rac*-**3** (109.7 mg, 59%) as a white solid. ^1^H-NMR (DMSO-*d*_6_, 500 MHz): δ = 8.15 (d, *J* = 2.3 Hz, 1H), 7.99 (ddd, *J* = 8.3 Hz, 5.8 Hz, 2.6 Hz, 1H), 7.08 (dd, *J* = 8.5 Hz, 2.9 Hz, 1H), 3.89 (t, *J* = 5.6 Hz, 1H), 3.52 (d, *J* = 4.1 Hz, 1H), 3.26–3.34 (m, 1H), 3.11 (bs, 1H), 3.07 (dd, *J* = 9.1 Hz, 5.6 Hz, 1H), 2.51–2.58 (m, 2H), 2.36–2.41 (m, 1H), 2.19–2.26 (m, 1H), 2.09 (dd, *J* = 13.3 Hz, 9.2 Hz, 1H), 1.72–1.79 (m, 1H). MS (ESI+): *m*/*z* 221.2 [M + H]^+^.

#### 3.2.2. (±)-3-*exo*,6-*exo*-6-(6-Fluoro-pyridin-3-yl)-8-aza-bicyclo[3.2.1]octan-3-ol ((±)-*exo*-3-hydroxy-flubatine, *rac*-**2a**) and (±)-3-*endo*,6-*exo*-6-(6-Fluoro-pyridin-3-yl)-8-aza-bicyclo[3.2.1]octan-3-ol ((±)-*endo*-3-hydroxy-flubatine, *rac*-**2b**)

Method (A): Deprotection of *dia*-**5**: Hydrobromic acid solution (33 wt % in acetic acid, 0.96 mL, 5.26 mmol, 25 eq.) was added to a solution of *dia*-**5** (1.3:1 mixture of diastereomers, 75 mg, 0.21 mmol) in 1 mL diethyl ether at 0 °C. The reaction mixture was stirred for 5 h at 0 °C. The solvent was removed *in vacuo*. Chloroform and water were added to the residue and the resulting mixture was washed with 1 M NaOH. The aqueous phase was extracted three times with chloroform. The combined organic phases were dried over sodium sulfate and the solvent was removed *in vacuo*. The diastereomeric products were separated by column chromatography (ethyl acetate:methanol = 3:1) to afford *rac*-**2a** (first eluting isomer, 10.7 mg, 23%) and *rac*-**2b** (second eluting isomer, 15.8 mg, 34%). ^1^H-NMR *rac*-**2b** (DMSO-*d*_6_, 500 MHz): δ = 8.14 (d, *J* = 2.2 Hz, 1H), 8.02 (ddd, *J* = 8.3 Hz, 5.8 Hz, 2.6 Hz, 1H), 7.08 (dd, *J* = 8.5 Hz, 2.9 Hz, 1H), 4.70 (bs, 1H), 3.90–3.99 (m, 2H), 3.64–3.72 (m, 1H), 3.49 (bs, 1H), 2.69 (dd, *J* = 12.5 Hz, 9.3 Hz, 1H), 1.83–2.01 (m, 3H), 1.66–1.78 (m, 2H). MS (ESI+): *m*/*z* 223.1 [M + H]^+^.

Method (B): Deprotection of *rac*-**5b**: *rac*-**5b** (74.4 mg, 0.21 mmol) was stirred in cyclohexene (1 mL) and ethanol (2 mL) until complete dissolution. The solution was placed under argon and Pd/C (10%) (44 mg, 0.04 mmol, 0.2 eq.) was added carefully. The reaction mixture was heated to reflux for 17 h. After cooling to rt the reaction mixture was filtered over celite. The filtration residue was washed with methanol and the solvent was removed *in vacuo*. The crude product was purified by column chromatography (methanol:ethyl acetate = 1:3 (1% trimethylamine)) to afford *rac*-**2b** (13 mg, 28%) as a white solid. As a byproduct the corresponding defluorinated compound, (24.6 mg, 58%) was isolated as white solid.

#### 3.2.3. (±)-3-*endo*,6-*exo*-6-(6-Fluoro-pyridin-3-yl)-3-hydroxy-8-aza-bicyclo[3.2.1]octane-8-carboxylic Acid Benzyl Ester (*rac*-**5b**)

l-Selectride solution (1 M in THF, 0.7 mL, 0.7 mmol, 3 eq.) was added over 10 min to a solution of *rac*-**3** (83 mg, 0.23 mmol, 1 eq.) in 1 mL THF at −78 °C. The reaction mixture was stirred for 2 h at −78 °C before 0.5 mL water was added and the mixture was allowed to warm to rt. A solution of sodium hydroxide (169 mg, 4.23 mmol) in 0.5 mL water and 0.48 mL hydrogen peroxide solution (30 wt % in water) were added successively and the mixture was stirred for 1 h at rt. The mixture was diluted with ethyl acetate and washed with water. The aqueous phase was extracted two times with ethyl acetate. The combined organic phases were washed with brine, dried over sodium sulfate and the solvent was removed *in vacuo*. The crude product was purified by column chromatography (hexane:ethyl acetate = 1:2) to afford *rac*-**5b** (81.5 mg, 97%) as a colourless oil. ^1^H-NMR (CDCl_3_, 500 MHz) two rotameric forms result in a doubling of some signals: δ = 8.03 (bs, 1H), 7.64–7.50 (m, 1H), 7.42–7.26 (m, 5H), 6.85–6.71 (m, 1H), 5.23–5.08 (m, 2H), 4.56–4.43 (m, 1H), 4.26–4.08 (m, 2H), 3.92–3.87 (m, 1H), 2.87–2.76 (m, 1H), 2.30–1.75 (m, 5H). MS (ESI+): *m*/*z* 357.0 (M + H)^+^; *m*/*z* 379.0 [M + Na]^+^.

### 3.3. Radiosynthesis of *(+)-[^18^F]**1***

(+)-[^18^F]**1** was obtained in radiochemical purity of 98% by nucleophilic substitution using cyclotron-produced [^18^F]fluoride (Cyclone-18/9 cyclotron, IBA, Louvain-la-Neuve, Belgium) as described for (−)-[^18^F]flubatine starting from the Boc-protected trimethylammonium iodide precursor [14].

### 3.4. Microsomal Incubations

(+)-**1** (1.2 mg) was dissolved in 1.0 mL water containing 5% acetonitrile. Twenty microliters of this solution was added to 562 μL water to provide a concentration of 0.2 mM. (−)-**1** (0.8 mg) was dissolved in 1.0 mL water containing 5% acetonitrile. Twenty microliters of this solution was added to 368 μL water to give a concentration of 0.2 mM. References were dissolved in identical manner. Testosterone (1.5 mg) was dissolved in 1.0 mL acetonitrile/water 2:3 (*v*/*v*) and used as a positive control. Nineteen microliters of this solution was added to 481 μL water to provide a concentration of 0.2 mM. For conducting microsome experiments the following instruments were used: BioShake iQ (QUANTIFOIL Instruments, Jena, Germany), Centrifuge 5424 (Eppendorf, Hamburg, Germany), Sample Concentrator DB-3D TECHNE (Biostep, Jahnsdorf, Germany). Incubations had a final volume of 250 μL and were performed in PBS (pH 7.4) at a final substrate concentration of 10 μM (for (+)-**1**, (−)-**1** and references) or 20 μM (for testosterone), a protein concentration of 1 mg/mL (for (+)- and (−)-**1**) or 2 mg/mL (for testosterone), and an NADPH concentration of 2 mM (for (+)- and (−)-**1**) or 4 mM (for testosterone). Conversion of the flubatine enantiomers was conducted in duplicate. A microsomal batch consisted for example of 12.5 μL of the prepared solution (0.2 mM) of (+)- or (−)-**1**, 12.5 μL of the protein solution (20 mg/mL) of MLM or HLM, and 25 μL NADPH (solution of 20 mM in PBS, pH 7.4). For the conversion of testosterone volumes of the ingredients were adjusted properly. Buffer, solutions of substrates, and liver microsomes were mixed and preincubated, as well as the solution of NADPH, by gentle shaking at 37 °C for 5 min. The reactions were initiated by adding the NADPH solution to the microsome-containing suspensions followed by gentle shaking at 37 °C. The incubations were terminated after 90 min by adding 1.0 mL of cold acetonitrile (−20 °C). After vigorous mixing for 30 s, the mixtures were stored at 4 °C for 5 min. Thereafter, centrifugation (14,000 rpm) was performed for 10 min and followed by concentration of the supernatants at 50 °C under a flow of nitrogen to provide residual volumes of 40–70 μL, which were reconditioned by adding water (acetonitrile/water 1:1 (*v*/*v*) for testosterone) to obtain samples of 100 μL, which were stored at 4 °C until analyzed. Beside the above mentioned batches, incubations without NADPH, microsomal protein, (+)- and (−)-**1**, or references, respectively, were analyzed as negative controls.

### 3.5. In Vivo Study of *(+)-[^18^F]**1*** in Mouse

The animal experiment was conducted under procedures approved by the respective State Animal Care and Use Committee and in accordance with the German Law for the Protection of Animals and the EU directive 2010/63/EU.

A female CD-1 mouse (32 g) received a tail vain injection of 250 MBq ((+)-[^18^F]**1** (specific activity >350 GBq/μmol) dissolved in 150 μL 5% ethanol/0.9% NaCl. At 30 min p.i. the animal was anaesthetized and killed by cervical dislocation. Brain, liver, and urine were removed. Whole blood was withdrawn by heart puncture, and plasma was obtained by centrifugation. Brain and liver were homogenized in 1–2 *v*/*w* 50 mM Tris-HCl, pH 7.4/4 °C using a Potter S Homogenizer (B. Braun, Melsungen, Germany). Homogenates, plasma and urine were extracted with ice-cold acetonitrile (1:4 *v*/*v*, −20 °C) using 4–12 aliquots of 125 μL, respectively. After shaking (3 min), cooling at 4 °C (5 min) and final shaking (3 min) the samples were centrifuged (7000 rpm, for liver 10,000 rpm, 4 °C, Centrifuge 5418, Eppendorf, Hamburg, Germany). Combined supernatants were concentrated using the Sample Concentrator DB-3D TECHNE (Biostep) at 75 °C under a flow of nitrogen to obtain residual volumes of less than 100 μL, which were reconditioned by adding water to obtain a final volume of 100 μL for investigation by radio-HPLC. For brain samples, the extraction procedure was repeated for each aliquot. For calculations of recoveries, aliquots of 25 μL were taken before and after centrifugation and, as well as the remaining residues, inspected by gamma counter (Wallac 1480, Wizard 3” PerkinElmer, Boston, MA, USA).

### 3.6. LC-MS Analyses

Analyses were performed on an Agilent 1260 Infinity Quaternary LC system (Agilent Technologies, Böblingen, Germany) coupled with a QTRAP 5500 hybrid linear ion-trap triple quadrupole mass spectrometer (AB SCIEX, Darmstadt, Germany). Data were acquired and processed using Analyst software (Version 1.6.1, AB SCIEX, Darmstadt, Germany). For further data processing Origin Pro 8.5.0G (OriginLab, Northampton, MA, USA) was used. For method I a Poroshell 120 EC-C18-column, 50 mm × 4.6 mm, 2.7 μm (Agilent Technologies, Böblingen, Germany) was used. The solvent system consisted of eluent A: aq. ammonium formate, 2.5 mM, pH 3 and eluent B: water/acetonitrile, 20:80 (*v*/*v*) (containing ammonium formate, 2.5 mM, pH 3). Gradient elution (% acetonitrile) at 25 °C and a flow rate of 1 mL/min: 0–9.0 min, 5%–37%; 9.0–9.1 min, 37%–80%; 9.1–11.0 min 80%; 11.0–11.1 min, 80%–5%; 11.1–14.0 min, 5%. For method II a Multospher 120 RP 18 AQ-5μ-column, 250 mm × 4.6 mm, 5 μm, including precolumn, 10 mm × 4 mm (CS Chromatographie Service, Langerwehe, Germany) was used. The solvent system consisted of eluent A: aq. ammonium formate, 25 mM, pH 3, and eluent B: aq. ammonium formate, 25 mM, pH 3/acetonitrile, 20:80 (*v*/*v*). Gradient elution (% acetonitrile) at a flow rate of 1.0 mL/min: 0–5.0 min, 5%; 5.0–40.0 min, 5%–30%; 40.0–41.0 min, 30%–80%; 41.0–51.0 min, 80%; 51.0–52.0 min, 80%–5%; 52.0–62.0 min, 5%.

The eluate was drained until 1.0 min (method I) or 5.0 min (method II) to avoid contamination by residual biological matrix. The mass spectrometer was operated in positive electrospray ionization mode and the following parameters were used unless otherwise stated: curtain gas (CUR) 40, collision gas (CAD) medium, ion spray voltage (IS) 4500, temperature (TEM) 600, ion source gas 1 (GS1) 70, ion source gas 2 (GS2) 60. The following scan types were applied:

- *Multiple reaction monitoring (MRM) scan type:* Substance-specific parameters were optimized for *rac***-1** delivered by syringe injection. Measurements were performed for the MRM transitions 207.1/110.0, 221.1/110.0, 223.1/110.0 and 239.1/110.0, applying the following parameters for method I: CAD high, scan time 80 ms, declustering potential (DP) 126, entrance potential (EP) 10, collision energy (CE) 37, collision cell exit potential (CXP) 10. For method II: CUR 30, CAD medium, IS 5500, TEM 500, GS1 50, GS2 50, scan time 100 ms, DP 136, EP 10, CE 44, CXP 14.
- *Q3 Multiple ion (MI) scan type:* Measurements were performed for *m*/*z* of 223.1, applying the following parameters: scan time 250 ms, DP 120, EP 10, CXP 37. For presentation purposes, chromatograms of blank samples were subtracted from chromatograms obtained initially, completed by final curve fitting.
- *Enhanced product ion (EPI) scan type:* By measuring solutions of *rac*-**2a** and *rac*-**2b**, supplied by a syringe pump, substance-specific parameters, allowing the detection of particular fragments of interest in EPI scan mode, were optimized. For measurements of the prepared incubation samples the following parameters were used: CAD high, IS 5500, TEM 650, GS1 60, GS2 60, products of *m*/*z* 221.1, 223.1, or 239.1 (respectively), dynamic fill time, DP 110, EP 10, CE 33, collision energy spread (CES) 0. In ion chromatograms obtained, a range of background was selected manually and subtracted from ranges of interest to result in EPI spectra as shown.

### 3.7. Radio-HPLC

Analyses were performed on a JASCO LC-2000 system (JASCO Labor- und Datentechnik, Gross-Umstadt, Germany) including UV/Vis detector UV-2070 (monitoring at 254 nm) online with a radioactivity flow detector GABI Star (raytest Isotopenmessgeräte, Straubenhardt, Germany) with NaI detector (2 × 2” pinhole, 16 mm × 30 mm). Chromatographic separations (method III) were achieved using a Multospher 120 RP 18 AQ-5μ-column, 250 mm × 4.6 mm, 5 μm, including precolumn, 10 mm × 4 mm (CS Chromatographie Service). The solvent system consisted of eluent A: aq. ammonium formate, 25 mM, pH 3/acetonitrile, 95:5 (*v*/*v*) and eluent B: aq. ammonium formate, 25 mM, pH 3/acetonitrile, 20:80 (*v*/*v*). Gradient elution (% acetonitrile) at a flow rate of 1.0 mL/min: 0–5 min, 5%; 5–26 min, 5%–20%; 26–27 min, 20%–80%; 27–37 min, 80%; 37–38 min, 80%–5%; 38–48 min, 5%.

## 4. Conclusions

Incubations of (+)- and (−)-flubatine with liver microsomes from mouse or human in the presence of NADPH showed low extent of metabolism. *In vitro* metabolites could be detected and most of their structures were elucidated by LC-MS/MS and comparison with reference compounds. After injection of (+)-[^18^F]flubatine ((+)-[^18^F]**1**) into mouse, analysis of radiometabolites and correlation with data obtained *in vitro* allowed the assignment of three monohydroxylated products. For identification of the main radiometabolite, phase II metabolism studies are needed.
